# Tertiary Syphilis

**Published:** 1846-05-01

**Authors:** 


*Tertiary Syphilis.* Female. Here we observe the existence of an ulcer upon either cheek, partially involving the ear. This woman confesses that she has had syphilis; if, however, she should stoutly deny that she had ever had the primary disease, I should unhesitatingly pronounce these ulcerations to be syphilitic. I should judge from the round shape of the ulcer, from its elevated edges, and from the peculiar glairy discharge. Also, from the rheumatic pain, of which she complains in the frontal region; this pain, she says, comes on after dinner, and is most severe in the afternoon. This is a valuable diagnostic mark. Syphilitic rheumatism may be distinguished from true rheumatism, by the fact, that the pain in acute rheumatism is most severe after the patient has retired, and is warmly covered up in bed; whereas, in the syphilitic form, it is more intense in the afternoon.

Treatment; in the tertiary form of syphilis, the iodide of potassium is the best remedy; it is good for nothing, however, in the primary and secondary forms of the disease. The formula which I use, is this: ℞. Iodid. potass. ℈ iv., Ext. conii. gr. xvj., Aquæ, ℥ iv.—M.

Dose, a table-spoonful twice a day; after a time it may be taken three times a day.—*Prof. Parker's Clinique. N. Y. Med. and Sur. Reporter.*

